# Acute sleep loss increases CNS health biomarkers and compromises the ability to stay awake in a sex-and weight-specific manner

**DOI:** 10.1038/s41398-022-02146-y

**Published:** 2022-09-10

**Authors:** Lieve T. van Egmond, Shervin Bukhari, Andrea Lessa Benedet, Nicholas J. Ashton, Elisa M. S. Meth, Alexander Boukas, Joachim Engström, Maria Ilemosoglou, Kaj Blennow, Henrik Zetterberg, Christian Benedict

**Affiliations:** 1 Department of Surgical Sciences, Functional Pharmacology and Neuroscience, Uppsala University, Uppsala, Sweden; 2 Department of Pharmaceutical Biosciences, Molecular Neuropharmacology (Sleep Science Laboratory), Uppsala University, Uppsala, Sweden; 3 Institute of Neuroscience and Physiology, Department of Psychiatry and Neurochemistry, The Sahlgrenska Academy at the University of Gothenburg, Mölndal, Sweden; 4 Clinical Neurochemistry Laboratory, Sahlgrenska University Hospital, Mölndal, Sweden; 5 Department of Neurodegenerative Disease, UCL Institute of Neurology, London, UK; 6 Dementia Research Institute at UCL, London, UK; 7 Hong Kong Center for Neurodegenerative Diseases, Hong Kong, China

**Keywords:** Diseases, Neuroscience

## Abstract

Night shift work impairs vigilance performance, reduces the ability to stay awake, and compromises brain health. To investigate if the magnitude of these adverse night shift work effects differs between sexes and weight groups, 47 men and women with either normal weight or obesity participated in one night of sleep and one night of total sleep loss. During the night of sleep loss, participants’ subjective sleepiness, vigilance performance, and ability to stay awake during 2-min quiet wake with eyes closed were repeatedly assessed. In addition, blood was collected in the morning after sleep loss and sleep to measure central nervous system (CNS) health biomarkers. Our analysis showed that women were sleepier during the night of sleep loss (*P* < 0.05) and spent more time in microsleep during quiet wake testing (*P* < 0.05). Finally, higher blood levels of neurofilament light chain, a biomarker of axonal damage, were found among women in the morning after sleep loss (*P* < 0.002). Compared with normal-weight subjects, those with obesity were more prone to fall asleep during quiet wake (*P* < 0.05) and exhibited higher blood levels of the CNS health biomarker pTau181 following sleep loss (*P* = 0.001). Finally, no differences in vigilance performance were noted between the sex and weight groups. Our findings suggest that the ability to stay awake during and the CNS health biomarker response to night shift work may differ between sexes and weight groups. Follow-up studies must confirm our findings under more long-term night shift work conditions.

## Introduction

Night shift work can impair job-related cognitive performance [1]. For example, a study among gas workers found that occupational performance errors peaked between 02:00 and 04:00 [2]. A separate study demonstrated that single-vehicle truck accidents reached their maximum between 01:00 and 07:00 [3]. Finally, emergency braking incidents caused by errors of response omission may be most prevalent between 03:00 and 06:00 [4]. In line with these observations, a study with 11 participants demonstrated that short periods of mock night shift work weakened performance on a wide range of cognitive tasks, including inhibitory control, sustained attention, diminished learning, and impaired visuospatial processing [5].

Besides the adverse effects on job-related cognitive performance, night shift work can have serious health complications [6], including adverse effects on central nervous system (CNS) health. For example, among 8059 nurses, those working night shifts for at least six years had a 50% higher dementia incidence than those engaged in night shifts for less than one year [7]. Similar findings were obtained in two cohorts from the Swedish Twin Registry in that night work was associated with 12% higher hazards to develop dementia than day work [8]. However, even short periods of night work may compromise brain health. For example, healthy young men without pre-existing neurological disorders showed increased blood levels of CNS health biomarkers after one night of wakefulness [9, 10]. For example, the evening-to-morning ratio for t-tau, a protein that is implicated in the development and progression of several brain disorders [11], was about 15% higher after one night of total sleep loss compared to sleep [9].

There are profound sex differences in the incidence and severity of neurodegenerative diseases, such as Alzheimer’s disease [12]. A meta-analysis including large population studies from multiple continents indicates that women are at greater risk of developing Alzheimer’s disease [13]. Obesity has emerged as an additional risk factor for developing dementia. For example, using data from the English Longitudinal Study of Ageing, people with obesity had a 34% elevated risk of dementia incidence compared to their normal-weight counterparts. Similar findings were found for people with central obesity [14].

Despite their association with brain health, it is unclear whether the female sex or having obesity alters the adverse brain health response to acute night shift work. Additionally, it has not been extensively researched whether sleepiness, the ability to stay awake, and vigilance performance—all highly relevant for occupational performance—differ by sex and weight status in a night shift setting. Thus, the present study measured CNS health biomarkers and cognitive performance during one night of in-laboratory sleep loss (mimicking a sedentary night shift setting) in men and women with either normal weight or obesity.

## Materials and methods

### Participants

Participants who reported no history of physical, psychiatric, or sleep-related diseases during the screening sessions were eligible for inclusion in the present study. All included participants had a habitual sleep duration of seven to nine hours between 22:00 and 08:00 during weekdays, did not habitually nap during the daytime, did not suffer from excessive daytime sleepiness (measured by the Epworth Sleepiness Scale [15]), did not report difficulties falling and staying asleep, did not report heavy snoring or other sleep-related breathing disturbances, did not report sleep movement disorders, did not report parasomnias, had no employment involving night or rotating shifts, and did not travel across time zones in the three weeks before nor during the study period. In addition, participants reported no use of regular medications. However, women included in the study took hormonal contraceptives to account for the potential confounding of the menstrual cycle. According to these criteria, 47 non-smoking adults (age 20–33 years; 21 women) were included. Men and women were characterized based on their biological sex (assessed via questionnaires). Obesity was defined as having a waist circumference >102 cm for men and >88 cm for women [16]. Normal weight was present when participants’ waist circumference was <94 cm for men and <80 cm for women [16]. As summarized in Table 1, men and women did not significantly differ in age, body mass index (BMI), waist circumference, and chronotype score (assessed by the Morningness Eveningness Questionnaire (MEQ); [17]). The weight groups were comparable for age and MEQ scores but not BMI and waist circumference.

**Table 1:** Cohort characteristics.

Characteristics	Subgroups				*P*	
	M	F	NW	OB	M vs. F	NW vs. OB
Participants, *n*	26	21	26	21	–	–
Male/Female, *n*	–	–	15/11	11/10	–	–
Age, years (mean ± SD)	25.1 ± 2.8	24.7 ± 2.9	24.8 ± 2.5	25.1 ± 3.2	0.63	0.73
BMI, kg/m^2^ (mean ± SD)	28.0 ± 6.7	27.2 ± 5.9	22.6 ± 2.0	33.9 ± 3.3	0.67	<0.001
Waist circumference, cm (mean ± SD)	95 ± 17	88 ± 17	78 ± 8	108 ± 9	0.19	<0.001
NW/OB, *n*	15/11	11/10	–	–	–	–
MEQ score (mean ± SD)	52.9 ± 7.9	51.3 ± 9.3	53.4 ± 9.5	50.7 ± 6.3	0.55	0.30

*P* < 0.05 was considered significant.

*BMI* body mass index, *F* female subgroup, *M* male subgroup, *MEQ* morningness eveningness questionnaire, *n* number, *NW* normal-weight subgroup, *OB* obese subgroup.

Subjects provided written informed consent before the study and were compensated for participation. The study was conducted according to the Declaration of Helsinki, and the Ethical Committee of Uppsala approved the experimental procedures (DNR2017/560). The experiment described herein is part of a more extensive study investigating the possible health consequences of sleep loss.

### Procedure

**Fig. 1:**
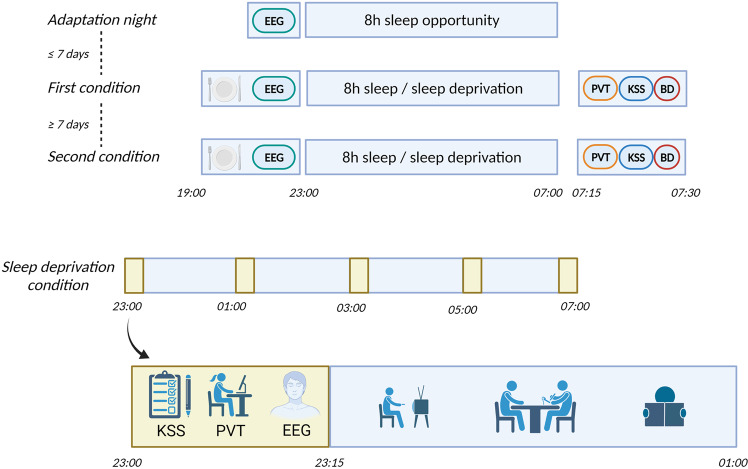
Experimental scheme. Order of experimental conditions. BD blood drawing, EEG electroencephalography, KSS Karolinska Sleepiness Scale, PVT Psychomotor vigilance task.

The study design and procedures are summarized in Fig. 1. Subjects participated in two counter-balanced experimental conditions separated by at least one week: one night of sleep vs. one night of total sleep loss. Experimental sessions of female participants took place outside their menstruation phase. In addition, within the week before the first experimental session, all subjects underwent an adaptation night to overcome the first-night effect [18]. In the week before each experimental condition, subjects also filled out sleep diaries specifying bed and rise times to ensure adherence to a regular sleep schedule. All experiments were performed between March 2018 and November 2020.

On experimental nights, subjects were served a standardized dinner upon arrival at the laboratory at 19:00. Participants could sleep between 23:00 and 07:00 (lights off period) in the sleep condition. Electroencephalography (EEG), electrooculography (EOG), and electromyography (EMG) were recorded by SOMNO HD following standard criteria (10–20 system [19]; SOMNOmedics GmbH, Randersacker, Germany). EOG recordings were collected from electrodes placed on the left upper and right lower outer canthi of the eyes. Sleep scoring was performed according to the American Academy of Sleep Medicine criteria [20]. In total, subjects slept 7 h and 12 min in the sleep condition. No significant difference in total sleep duration between participants who started with the sleep condition and those who had their sleep condition about one week later after the wake condition were found (mean ± SD: 435 ± 35 min vs. 432 ± 26 min; *P* = 0.769 as derived from an independent Student’s *t*-test). The SOMNO HD pulse oximeter was also used to estimate the number of desaturation events (Oxygen Desaturation Index; ODI) per hour during sleep (defined as a decrease in blood oxygen saturation greater than 3% from baseline [21]). At 07:15 in the morning after sleep, subjects were administered a psychomotor vigilance test (PVT) and Karolinska Sleepiness Scale (KSS) at ~07:15, i.e., about 15 min upon awakening, to avoid sleep inertia effects.

During the night of sleep loss, participants could spend their time with sedentary activities, e.g., watching movies and reading books, but had to stay in the laboratory rooms with the experimenter under normal light conditions (~500 lux). However, no food intake, including caffeinated beverages, was allowed. Participants took part in a test battery at 23:00, 01:00, 03:00, 05:00, and 07:00. This battery included the KSS, PVT, and a wake maintenance task. In addition, at 07:15, subjects were again administered the KSS. Blood was drawn at 07:30 via venipuncture in both experimental conditions to compare possible differences in CNS health biomarkers.

### KSS

The KSS [22] is a nine-point ordinal scale that measures subjective sleepiness as follows: “extremely alert”—one point, “alert”—three points, “neither alert nor sleepy”—five points, “sleepy—but no difficulty staying awake”—seven points, and “extremely sleepy—fighting sleep”—nine points.

### PVT

The PVT is a visual reaction time test to assess objective vigilance [23]. We used a three min version as validated for and sensitive to sleep deprivation [24]. The task was administered using the Psychology Experiment Building Language (Versions 0.14 & 2.1; [25]). Participants were instructed to focus their attention on a fixation cross and press the spacebar on a computer keyboard as quickly as possible upon the appearance of the stimulus (a red dot, 400 msec presentation time). Stimulus onset asynchrony varied between two and twelve sec. No performance feedback was provided. Trials with reaction times <100 msec were counted as false starts, and trials with reaction times ≥500 msec were defined as lapses. False starts and lapses were removed before calculating the mean reaction time. Outcome variables derived from the PVT were mean reaction time and the number of lapses.

### Wake maintenance task

To measure the ability of participants to stay awake during the night of the sleep loss condition, we administered a wake maintenance task, which adopted the setting of the Karolinska Drowsiness Test [22]. Briefly, after five min of quiet sitting with open eyes, participants closed their eyes for two min with their heads on a chin rest. The general instruction was to stay awake quietly but not to fall asleep.

Following the acquisition, 120 sec of EEG data from eyes closed trials were visually inspected in 30-sec epochs for the occurrence of sleep and microsleep events. Microsleep events were defined as a period of at least three sec during which occipital alpha wave activity (8–13 Hz) subsided, and theta activity replaced the background alpha [26]. If the microsleep episode exceeded 15 sec, it was scored as sleep according to standard guidelines [20]. The following variables derived from the wake maintenance task were used for the analysis: (a) the total duration of microsleep episodes; and (b) the total duration of sleep episodes.

### CNS health biomarkers

Blood from 40 participants (25 men; 15 women; 25 subjects with normal weight; 15 subjects with obesity) was successfully collected into PSTII tubes (BD Sweden, Stockholm), in the morning via venipuncture. Following centrifugation (1300 rcf, 10 min, 4 °C), and aliquoting of the supernatant, samples were stored at −80 °C until analysis. Tau protein phosphorylated at amino acid 181 and 231, respectively, were measured using in-house Single molecule array (Simoa) assays as previously described [27, 28]. Aβ40 and Aβ42, glial fibrillary acidic protein (GFAP), and neurofilament light chain (NfL) concentrations were measured using the Quanterix Neurology 4-Plex E Advantage Kit (Quanterix, Billerica, Massachusetts). All measurements were performed in one round of experiments on the Single molecule array (Simoa) ND-X Analyser (Quanterix, Billerica, Massachusetts). Intra-assay coefficients of variation were below 8% (majority below 5%).

### Statistical analysis

Data were analyzed using IBM SPSS Statistics 26 (SPSS Inc. Chicago, IL, USA). The normal distribution of variables was assessed by visual inspection and the Shapiro–Wilk test. Skewed data were log-transformed to approach normality and improve model fit. Baseline differences (i.e., at 23:00) between sexes and weight groups were analyzed using independent *t*-tests. Potential differences in the ratings, vigilance, and blood biomarkers between the sleep and sleep loss conditions in the whole sample were examined with paired t-tests.

To account for baseline differences in the PVT between sex and weight groups, the individual mean reaction time at 01:00, 03:00, 05:00, and 07:00 was expressed as a percentage of the mean reaction time at 23:00 (defined as the baseline). As most participants had zero events at 23:00 for lapses during the PVT and microsleep and sleep episodes on the wake maintenance task, we calculated absolute differences to the baseline for each participant. The CNS health biomarkers exhibited large inter-individual variance. Thus, we divided blood levels measured in the morning after sleep loss by those measured after sleep. Given that the KSS scale has an ordinal structure, this variable was not baseline-adjusted.

To investigate whether sleepiness, vigilance, and the ability to stay awake vary by sex and weight status during the night of the sleep loss condition, we ran generalized linear mixed models (GLMM), including the within-subjects factor TIME (01:00, 03:00, 05:00, and 07:00) and the between-subjects factors SEX and WEIGHT as fixed factors. As skewed data were log-transformed, we used a normal probability distribution with an identity link function in all GLMM models. In the case of blood biomarkers, we applied generalized linear models (GLM), using SEX and WEIGHT as fixed between-subjects factors. The time in bed (TIB) in the night preceding the experimental wake condition (mean ± SD: men, 484 min ± 71 min; women, 523 min ± 51 min; with normal weight, 485 min ± 60 min; and with obesity: 520 min ± 70 min) was included as a covariate in all GLMM and GLM analyses. We included this covariate to reduce the impact of day-to-day variability in sleep schedules between participants on our study outcomes. Interactions between the between-subject factors SEX and WEIGHT were not modeled. Post-hoc testing was performed using pairwise t-tests with LSD adjustment for multiple comparisons. Correlation analyses were performed using Spearman’s rho tests as the data was not normally distributed. Unless otherwise specified, data are reported as estimated marginal means [95%–CI]. *P* < 0.05 was considered significant.

## Results

### KSS

**Fig. 2:**
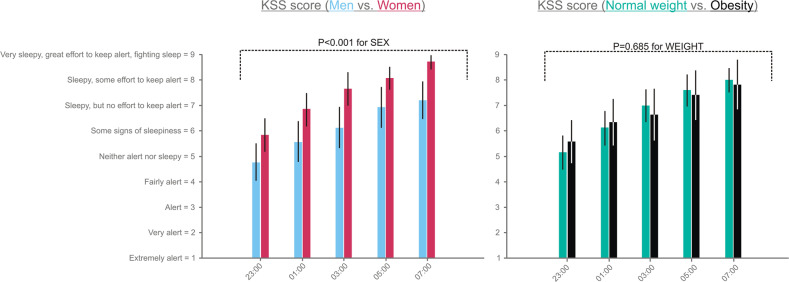
Subjective sleepiness during the sleep loss night. Values are shown as estimated marginal means [95%-CI] as derived from generalized linear mixed models. Baseline (i.e., at 23:00) Karolinska Sleepiness Scale (KSS) scores (mean ± SD): men, 4.8 ± 1.9; women: 5.7 ± 1.5; with normal weight: 5.1 ± 1.6; and with obesity: 5.4 ± 1.9. *P* < 0.05 was considered significant.

During the night of sleep loss, subjective sleepiness increased by 2.6 points between 23:00 and 07:00 (*P* < 0.001 for TIME). As summarized in Fig. 2, KSS scores were, on average, 1.3 points higher in women than men (7.4 [7.0, 7.8] vs. 6.1 [5.8, 6.4], *P* < 0.001 for SEX). However, the SEX*TIME interaction did not reach significance (*P* = 0.963). Furthermore, whereas both weight groups showed an apparent increase in sleepiness during sleep loss (Fig. 2), no main or interaction effects were found (*P* = 0.685 for WEIGHT; *P* = 0.841 for WEIGHT*TIME). When comparing sleep loss with sleep, at 07:15, subjects felt sleepier in the morning after sleep loss than after sleep (7.5 [7.0, 8.0] vs. 3.3 [2.8, 3.9], *P* < 0.001, paired *t*-test).

### PVT

**Fig. 3:**
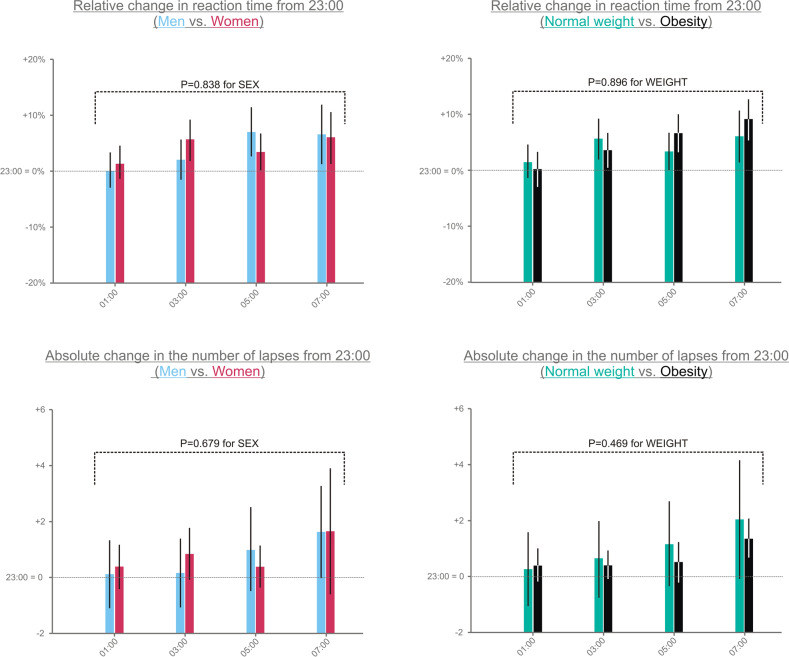
Performance on the psychomotor vigilance task. Values are shown as estimated marginal means [95%-CI] as derived from generalized linear mixed models. Baseline (i.e., at 23:00) reaction time (mean ± SD): men, 314 ± 43; women: 330 ± 45; with normal weight: 323 ± 50; and with obesity: 318 ± 35. Number of lapses at baseline (mean ± SD): men, 1.6 ± 3.3; women: 1.8 ± 4.3; with normal weight: 2.5 ± 4.8; and with obesity: 0.6 ± 0.9. *P* < 0.05 was considered significant.

The reaction time increased significantly throughout the night of the sleep loss (e.g., +6% longer at 07:00 compared to 23:00, *P* = 0.016 for TIME). However, the increase in reaction time did not differ between sexes (men vs. women: +3.7% [1.9%, 5.6%] vs. +4.1% [1.6%, 6.5%] from baseline, *P* = 0.838 for SEX; Fig. 3). Likewise, the reaction time did not differ between participants with normal weight and obesity (+4.0% [2.1%, 5.9%] vs. +3.8% [1.5%, 6.2%] from baseline, *P* = 0.896 for WEIGHT; Fig. 3). Lastly, no significant interactions of SEX and WEIGHT with TIME were found (*P* ≥ 0.381).

As shown in Fig. 3, the number of lapses did not differ by TIME (*P* = 0.303), SEX (men vs. women: +0.7% [0.1%, 1.4%] vs. +0.9% [0.1%, 1.8%] from baseline, *P* = 0.679), and WEIGHT (normal weight vs. obese; 1.0% [0.4%, 1.7%] vs. 0.7% [−0.1%, −1.5%] from baseline, *P* = 0.469; *P* ≥ 0.802 for all interactions with TIME).

Finally, in line with previous findings [24], the reaction time and the number of lapses were significantly higher in the morning following sleep loss than after sleep (reaction time: 340 ms [325 ms, 354 ms] vs. 312 ms [300 ms, 323 ms]; and the number of lapses: 3.1 [1.5, 4.7] vs. 1.6 [0.5, 2.6], *P* ≤ 0.014, paired *t*-tests).

### Wake maintenance task

**Fig. 4:**
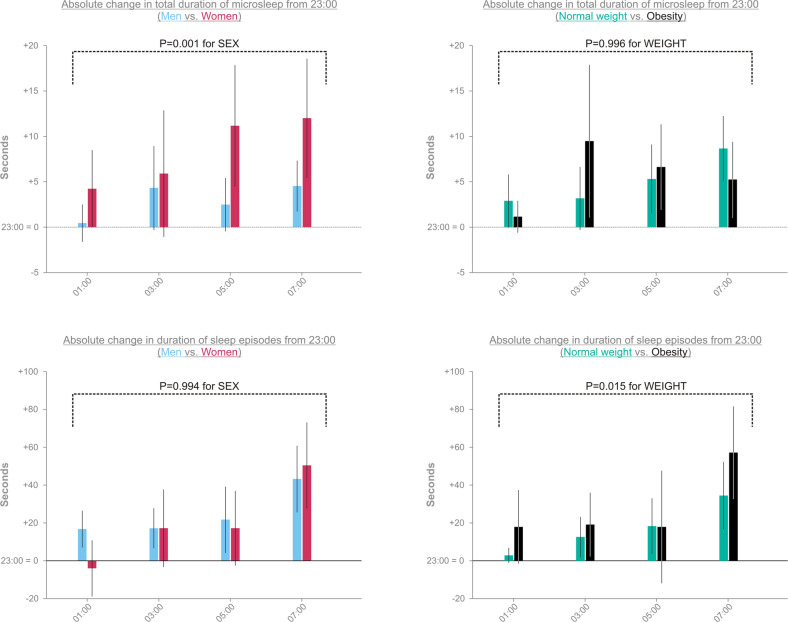
Duration of microsleep and sleep episodes during the wake maintenance task. Values are shown as estimated marginal means [95%-CI] as derived from generalized linear mixed models. Total duration of microsleep at baseline (i.e., at 23:00; mean ± SD): men, 0.7 s ± 2.3 s; women: 0.3 s ± 1.4 s s; with normal weight: 0.8 s ± 2.5 s; and with obesity: 0.2 s ± 0.7 s. Total duration of sleep episodes at baseline (mean ± SD): men, 8.8 s ± 25.7 s; women: 11.1 s ± 28.7 s; with normal weight: 4.8 s ± 18.7 s; and with obesity: 16.3 s ± 34.0 s. *P* < 0.05 was considered significant.

As shown by the wake maintenance task testing the ability to stay awake during 2-min of quiet wake with closed eyes, the duration of microsleep and sleep episodes increased throughout the sleep loss condition (+7.7 s microsleep duration at 07:00 compared to 23:00, *P* = 0.01; +45.5 sec sleep duration at 07:00 compared to 23:00, *P* < 0.001). Furthermore, as shown in Fig. 4, a main effect of SEX was found (*P* = 0.001) in that women spent more time in microsleep than men (+8.1 s [1.3 s, 5.6 s] vs. +2.9 s [1.2 s, 4.6 s] from baseline). In contrast, no sex differences in sleep duration were observed (+23.5 sec [13.3 s, 33.8 s] from baseline for women vs. +23.5 s [16.6 s, 30.4 s] from baseline for men, *P* = 0.994 for SEX).

Compared with normal-weight subjects, those with obesity struggled more with staying awake during the wake maintenance task, as indicated by the difference in the total sleep duration between the weight groups (normal-weight vs. obese group: +16.6 sec [9.3 s, 23.8 s] vs. +30.4 s [20.9 s, 40.0 s] from baseline, *P* = 0.015; Fig. 4). In contrast, no WEIGHT effects on the total duration of microsleep were found (normal-weight vs. obese group: +5.5 s [3.7 s, 7.3 s] vs. +5.5 s [3.1 s, 7.8 s] from baseline, *P* = 0.996; Fig. 4). Overall, none of the modeled interactions reached significance (*P* ≥ 0.166).

### CNS health biomarkers

Ratios and raw data for each CNS health biomarker can be found in Supplemental Tables S1, S2. Blood concentrations of pTau181 were about 25% higher after sleep loss than after sleep (*P* = 0.003). In contrast, blood levels of pTau231, NfL, Aβ40, Aβ42, and GFAP did not differ between the sleep and sleep loss conditions (*P* ≥ 0.054).

**Fig. 5:**
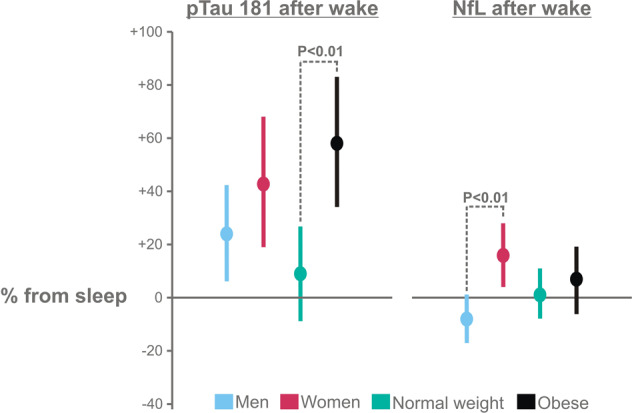
Morning blood concentrations of the CNS health biomarkers NfL and pTau181 after sleep loss expressed as fold change from blood concentrations measured after sleep. Values are shown as estimated marginal means [95%-CI] as derived from general linear models. *P* < 0.05 was considered significant. pTau Tau protein, NfL neurofilament light chain.

When using individual ratios for each CNS health biomarker, we found that the increase in pTau181 following sleep loss was more pronounced for participants with obesity than those with normal weight (1.58 [1.34, 1.83] vs. 1.09 [0.91, 1.27] fold change from sleep, *P* = 0.001 for WEIGHT; Fig. 5). Finally, relative blood levels of NfL measured in the morning after sleep loss were higher among women than men (1.16 [1.04, 1.28] vs. 0.92 [0.83, 1.01] fold change from sleep, *P* = 0.002 for SEX; Fig. 5).

To account for possible carrying-over effects after one night of acute sleep loss on CNS health biomarkers measured about one week later in the sleep condition, we re-ran the analysis using the order of the experimental conditions (i.e., first wake, then sleep, or vice versa) as a possible confounder. However, the results remained the same (data not shown).

### Correlational analysis

Using correlational analysis, we found no association between the cumulative duration of microsleep episodes during the wake condition and the sleep loss-to-sleep ratio for NfL in women (Spearman’s rho = −0.469, *P* = 0.124). Additionally, variance in the sleep loss-to-sleep ratio for NfL among women was not significantly explained by the cumulative KSS score measured during the wake condition (Spearman’s rho = −0.208, *P* = 0.408).

Among those with obesity, the cumulative time asleep during the wake maintenance task was not significantly associated with the sleep loss-to-sleep ratio for pTau181 (Spearman’s rho = −0.408, *P* = 0.212). Although the ODI was generally higher among participants with obesity (vs. normal-weight subjects, mean ± SD: 4.8 ± 5.2 vs. 1.4 ± 1.5, *P* = 0.02 as derived from an independent Student’s t-test), no significant association between the sleep loss-to-sleep ratio for pTau181 and the ODI was found (Spearman’s rho = 0.278, *P* = 0.117).

## Discussion

The present study investigated if changes in subjective sleepiness, the ability to stay awake, vigilance performance, and CNS health blood biomarkers during one night of sleep loss differ between sex and weight groups. During the night of in-laboratory sleep loss, women reported greater sleepiness and struggled more with staying awake, as indicated by a more extended time spent in microsleep during quiet wake testing. Finally, women had higher blood levels of NfL, a biomarker of axonal damage [28], after sleep loss relative to sleep. When focusing on the weight status of the included participants, we found that those with obesity were more prone to fall asleep than normal-weight subjects during the night of sleep loss. In addition, they exhibited higher levels of pTau181 following sleep loss relative to sleep. This biomarker rises in diseases hallmarked by neurodegeneration and predicts brain atrophy in aging [29].

In recent years, increasing evidence suggests that night shift work can lead to long-term cognitive impairment. For example, a French study reported lower global cognitive test scores for people with more than ten years of shift work history [30]. Furthermore, a Swedish study revealed that current and recent former shift workers (defined as shift work during the past five years) performed worse on an executive cognitive function task [31]. These epidemiological observations have been corroborated by experimental findings [32, 33]. A meta-analysis found that a lack of sleep, ranging from 24 to 48 h, significantly reduced performance in multiple cognitive domains, including reaction time and attentional lapses [33]. Interestingly, the effects of sleep loss on cognitive functions may differ between men and women. For example, a study involving 34 participants (18 women) found that the circadian nadir in cognitive performance coinciding with the night was more pronounced for women [34]. However, the study did not observe sex differences in subjective sleepiness during the sleep loss night [34]. In the present study, we found that women felt sleepier but showed no difference in cognitive performance (here measured by the PVT) compared to men. One explanation for the discrepancy in results could be that we only included women taking contraceptive medication. In contrast, half of the women in the previous study were not on contraceptives [34]. As shown by a separate experiment, the vulnerability to attentional failure due to nocturnal wakefulness can vary considerably across the menstrual cycle [35].

The association between night shift work and excessive weight is well established. For example, a meta-analysis found a 1.2-fold greater risk of overweight or obesity among night shift than daytime workers, especially for developing abdominal obesity [36]. However, even though obesity is negatively associated with different domains of cognitive functioning [37, 38], it is unknown whether obesity impairs a person’s ability to stay awake and be alert throughout one night of wakefulness. As suggested by our study, participants with obesity showed a similar rise in sleepiness and drop in vigilance as normal-weight subjects across the sleep loss night. However, participants with obesity struggled more to stay awake than those with normal weight during the wake maintenance task. The observation that the risk to fall asleep during quiet wake was more pronounced among those with obesity indicates that nocturnal wake periods with a lack of cognitive demand may be more challenging for people with obesity.

Women are at significantly greater risk of developing Alzheimer’s disease [12]. Furthermore, obesity appears to be associated with faster brain aging [39]. Thus, we examined whether changes in CNS health blood biomarkers following one night of sleep loss differed between sex and weight groups. Our analysis showed that women exhibited higher blood levels of NfL after sleep loss relative to sleep, whereas no such difference occurred in men. Furthermore, as indicated by greater sleepiness and higher abundance of microsleep episodes, the sleep pressure during the mock night shift was greater among women than men. Noteworthy, until mid-life, women report longer ideal sleep duration than men [40], suggesting that forced wakefulness may represent a more significant burden for the female brain, with possible negative implications for brain health. However, no correlations were found between the relative increase in NfL after sleep loss and the cumulative KSS score. Likewise, the total duration of microsleeps occurring during the night of forced wakefulness did not predict variance in the relative increase in NfL among women.

We found that the night shift session was more stressful for the brain of participants with obesity, as indicated by a more pronounced relative increase in pTau181 after sleep loss. Those with obesity also struggled more with staying awake, as they were more prone to fall asleep during the wake maintenance task; however, the relative increase in pTau181 after sleep loss did not correlate with this sleep measure. There are several potential explanations for why participants with obesity may have experienced more sleep pressure during the wake condition. First, increased sleep pressure may be due to less restorative sleep, e.g., caused by sleep-disordered breathing [41]. While we did not measure sleep-disordered breathing on the nights preceding the experimental wake condition, we found a greater ODI in those with obesity on the experimental sleep night. Thus, participants with obesity may have started the night shift session with greater sleep debt, thereby imposing a more significant burden on CNS health. However, no correlation between the ODI and the relative increase in pTau181 was seen among the group with obesity. Another explanation for increased sleep pressure due to forced wakefulness could be low-grade inflammation associated with obesity. Excessive adiposity is characterized by increased secretion of pro-inflammatory cytokines from the adipose tissue, including tumor necrosis factor-alpha. This cytokine increases sleep pressure in humans [42, 43].

One possible mechanism for the observed rise in pTau181 and NfL due to sleep loss could be reduced activity of the glymphatic system, a waste clearance system in the brain that is highly active during nighttime sleep [44]. Notwithstanding the underlying mechanism, based on our findings, future studies should investigate whether more extended periods of night shift work are associated with a more significant CNS burden among women and those with obesity.

A significant strength of our study is that the in-laboratory experimental procedure reduced bias due to external factors (e.g., consumption of caffeinated beverages during the night or differences in the ambient light conditions). However, only young adults who had no previous experience with regular night shift work, and only women on hormonal contraceptives, were included in our study. Furthermore, it is unclear whether natural inter- and intraindividual variations in sex hormones (including testosterone) alter the effects of night shift work on brain health and cognitive performance in men and women. Thus, future studies are needed to unravel the possible consequences of night shift work on brain health and cognitive performance in more diverse cohorts. Another limitation is that CNS health biomarkers were only measured once in the morning after each experimental session. It remains to be investigated whether blood concentrations of pTau231, NfL, Aβ40, Aβ42, and GFAP may rise in response to acute sleep loss at other times of the day.

In the wake condition, participants did not eat and were mainly sedentary. Hence, it is unclear whether the adverse effects of night shift work on the herein investigated outcomes are affected by factors such as eating during a night shift, naps before and during the night shift, and the level of physical and cognitive occupational demand. Finally, factors like light exposure before and during the night may affect how alertness, cognitive performance, and brain health are affected by night shift work. For example, continuous blue light exposure during nocturnal driving has been shown to be promising in mitigating sleepiness at the wheel [45].

## Conclusions

Our findings suggest that women and people with obesity may struggle more with unintentionally falling asleep during a night of sleep loss. In addition, the rise of the CNS health biomarkers NfL in women and pTau181 in participants with obesity suggests that these groups may be more susceptible to the adverse effects of night shift work on brain health. However, whether longer periods of night shift work lead to irreversible neurodegeneration remains unclear.

## Supplementary information

Supplemental Material 5

## References

[1] Mitler MM, Carskadon MA, Czeisler CA, Dement WC, Dinges DF, Graeber RC (1988). Catastrophes, sleep, and public policy: Consensus report. Sleep.

[2] Bjerner B, Holm A, Swensson A (1955). Diurnal variation in mental performance; a study of three-shift workers. Br J Ind Med.

[3] Miller JC, Mackie RR, Effects of hours of service, regularity of schedules and cargo loading on truck and bus driver fatigue. Hum Factors Res Inc Tech Rep No 1765-F. Published online January 1, 1978.

[4] Hildebrandt G, Rohmert W, Rutenfranz J (1974). 12 and 24 h Rhythms in error frequency of locomotive drivers and the influence of tiredness. Int J Chronobiol.

[5] Hart CL, Haney M, Vosburg SK, Comer SD, Gunderson E, Foltin RW (2006). Modafinil attenuates disruptions in cognitive performance during simulated night-shift work. Neuropsychopharmacology.

[6] Kecklund G, Axelsson J. Health consequences of shift work and insufficient sleep. BMJ. 2016;355;i5210. 10.1136/bmj.i5210 27803010

[7] Jørgensen JT, Hansen J, Westendorp RGJ, Nabe-Nielsen K, Stayner LT, Simonsen MK (2020). Shift work and incidence of dementia: A Danish Nurse Cohort study. Alzheimer’s Dement.

[8] Bokenberger K, Sjölander A, Dahl Aslan AK, Karlsson IK, Åkerstedt T, Pedersen NL (2018). Shift work and risk of incident dementia: a study of two population-based cohorts. Eur J Epidemiol.

[9] Benedict C, Blennow K, Zetterberg H, Cedernaes J (2020). Effects of acute sleep loss on diurnal plasma dynamics of CNS health biomarkers in young men. Neurology.

[10] Benedict C, Cedernaes J, Giedraitis V, Nilsson EK, Hogenkamp PS, Vågesjö E (2014). Acute sleep deprivation increases serum levels of neuron-specific enolase (NSE) and S100 calcium-binding protein B (S-100B) in healthy young men. Sleep.

[11] Shahim P, Zetterberg H (2022). Neurochemical Markers of Traumatic Brain Injury: Relevance to Acute Diagnostics, Disease Monitoring, and Neuropsychiatric Outcome Prediction. Biol Psychiatry.

[12] Laws KR, Irvine K, Gale TM (2018). Sex differences in Alzheimer’s disease. Curr Opin Psychiatry.

[13] Gao S, Hendrie HC, Hall KS, Hui S (1998). The relationships between age, sex, and the incidence of dementia and Alzheimer disease: a meta-analysis. Arch Gen Psychiatry.

[14] Ma Y, Ajnakina O, Steptoe A, Cadar D (2020). Higher risk of dementia in English older individuals who are overweight or obese. Int J Epidemiol.

[15] Johns MW (1991). A new method for measuring daytime sleepiness: the Epworth sleepiness scale. Sleep.

[16] American College of Cardiology/American Heart Association Task Force on Practice Guidelines, Obesity Expert Panel, 2013. Executive summary: Guidelines (2013) for the management of overweight and obesity in adults: a report of the American College of Cardiology/American Heart Association Task Force on Practice Guidelines and the Obesity Society published by the Obesity Society and American College of Cardiology/American Heart Association Task Force on Practice Guidelines. Based on a systematic review from the The Obesity Expert Panel, 2013. Obesity (Silver Spring). 2014 Jul;22:S5-S39. 10.1002/oby.20821 24961825

[17] Horne JA, Ostberg O (1976). A self assessment questionnaire to determine Morningness Eveningness in human circadian rhythms. Int J Chronobiol.

[18] Tamaki M, Bang JW, Watanabe T, Sasaki Y (2016). Night watch in one brain hemisphere during sleep associated with the first-night effect in humans. Curr Biol.

[19] Jasper HH. The Ten Twenty Electrode System: International Federation of Societies for Electroencephalography and ClinicalNeurophysiology. Am J EEG Technol. 1958;10:371–375.

[20] Berry RB, Brooks R, Gamaldo CE, Harding SM, Lloyd RM, Marcus CL and Vaughn BV for the American Academy of Sleep Medicine. The AASM Manual for the Scoring of Sleep and Associated Events: Rules, Terminology and Technical Specifications, Version 2.2. www.aasmnet.org. Darien, Illinois: American Academy of Sleep Medicine, 2015.

[21] Temirbekov D, Güneş S, Yazıcı ZM, Sayın İ (2018). The ignored parameter in the diagnosis of obstructive Sleep Apnea Syndrome: The Oxygen Desaturation Index. Turk Arch Otorhinolaryngol.

[22] Åkerstedt T, Gillberg M (1990). Subjective and objective sleepiness in the active individual. Int J Neurosci.

[23] Dinges DF, Powell JW (1985). Microcomputer analyses of performance on a portable, simple visual RT task during sustained operations.. Behav Res Methods Instrum Comput.

[24] Basner M, Mollicone D, Dinges DF (2011). Validity and sensitivity of a brief psychomotor vigilance test (PVT-B) to total and partial sleep deprivation. Acta Astronaut.

[25] Mueller ST, Piper BJ (2014). The Psychology Experiment Building Language (PEBL) and PEBL Test Battery. J Neurosci Methods.

[26] Boyle LN, Tippin J, Paul A, Rizzo M (2008). Driver performance in the moments surrounding a microsleep. Transp Res Part F Traffic Psychol Behav.

[27] Ashton NJ, Pascoal TA, Karikari TK, Benedet AL, Lantero-Rodriguez J, Brinkmalm G (2021). Plasma p-tau231: a new biomarker for incipient Alzheimer’s disease pathology. Acta Neuropathol.

[28] Khalil M, Pirpamer L, Hofer E, Voortman MM, Barro C, Leppert D (2020). Serum neurofilament light levels in normal aging and their association with morphologic brain changes. Nat Commun.

[29] Tissot C, L Benedet A, Therriault J, Pascoal TA, Lussier FZ, Saha-Chaudhuri P, et al. Plasma pTau181 predicts cortical brain atrophy in aging and Alzheimer’s disease. Alzheimer’s Res Ther. 2021;13:69. 10.1186/s13195-021-00802-x PMC8008680 33781319

[30] Marquié JC, Tucker P, Folkard S, Gentil C, Ansiau D (2015). Chronic effects of shift work on cognition: Findings from the VISAT longitudinal study. Occup Environ Med.

[31] Titova OE, Lindberg E, Elmståhl S, Lind L, Schiöth HB, Benedict C (2016). Association between shift work history and performance on the trail making test in middle-aged and elderly humans: The EpiHealth study. Neurobiol Aging.

[32] Van Dongen HPA, Maislin G, Mullington JM, Dinges DF (2003). The cumulative cost of additional wakefulness: Dose-response effects on neurobehavioral functions and sleep physiology from chronic sleep restriction and total sleep deprivation. Sleep.

[33] Lim J, Dinges DF (2010). A meta-analysis of the impact of short-term sleep deprivation on cognitive variables. Psychol Bull.

[34] Santhi N, Lazar AS, McCabe PJ, Lo JC, Groeger JA, Dijk DJ (2016). Sex differences in the circadian regulation of sleep and waking cognition in humans. Proc Natl Acad Sci USA.

[35] Vidafar P, Gooley JJ, Burns AC, Rajaratnam SMW, Rueger M, et al. Increased vulnerability to attentional failure during acute sleep deprivation in women depends on menstrual phase. Sleep. 2018;41:zsy098. 10.1093/sleep/zsy098 PMC6093460 29790961

[36] Sun M, Feng W, Wang F, Li P, Li Z, Li M (2018). Meta-analysis on shift work and risks of specific obesity types. Obes Rev.

[37] Tanaka H, Gourley DD, Dekhtyar M, Haley AP (2020). Cognition, brain structure, and brain function in individuals with obesity and related disorders. Curr Obes Rep.

[38] Prickett C, Brennan L, Stolwyk R (2015). Examining the relationship between obesity and cognitive function: A systematic literature review. Obes Res Clin Pr.

[39] O’Brien PD, Hinder LM, Callaghan BC, Feldman EL (2017). Neurological consequences of obesity. Lancet Neurol.

[40] Tonetti L, Fabbri M, Natale V (2008). Sex difference in sleep-time preference and sleep need: a cross-sectional survey among Italian pre-adolescents, adolescents, and adults. Chronobiol Int.

[41] Mediano O, Barceló A, de la Peña M, Gozal D, Agustí A, Barbé F (2007). Daytime sleepiness and polysomnographic variables in sleep apnoea patients. Eur Respir J.

[42] Ouchi N, Parker JL, Lugus JJ, Walsh K (2011). Adipokines in inflammation and metabolic disease. Nat Rev Immunol.

[43] Krueger JM, Clinton JM, Winters BD, Zielinski MR, Taishi P, Jewett KA (2011). Involvement of cytokines in slow wave sleep. Prog Brain Res.

[44] Hauglund NL, Pavan C, Nedergaard M (2020). Cleaning the sleeping brain—the potential restorative function of the glymphatic system. Curr Opin Physiol.

[45] Taillard J, Capelli A, Sagaspe P, Anund A, Akerstedt T, Philip P (2012). In-car nocturnal blue light exposure improves motorway driving: a randomized controlled trial. PLoS One.

