# Supplementary material for: Acute sleep loss increases CNS health biomarkers and compromises the ability to stay awake in a sex-and weight-specific manner
Source: Transl Psychiatry. 2022 Sep 10;12:379. doi: 10.1038/s41398-022-02146-y (PMC9464235; doi:10.1038/s41398-022-02146-y)
Supplement: Supplementary file 1 — Supplemental Material 5 [file 41398_2022_2146_MOESM1_ESM.docx]

**Supplement 1, Table S1. CNS health biomarkers in blood following sleep loss expressed as fold change from blood levels measured after sleep.**

| **Factor** | **Entire cohort^A^** | **Sex groups^B^** | | **Weight groups^B^** | |
| --- | --- | --- | --- | --- | --- |
|  | **(N=40)** | **Men (N=25)** | **Women (n=15)** | **NW (n=25)** | **OB (n=15)** |
| **pT231** | 1.10 [0.99, 1.20] | 1.15 [1.02, 1.28] | 1.01 [0.83, 1.18] | 1.08 [0.95, 1.21] | 1.07 [0.90, 1.25] |
| **pT181** | **1.25 [1.08, 1.43] **** | 1.24 [1.06, 1.42] | 1.43 [1.19, 1.68] | 1.09 [0.91, 1.27] | **1.58 [1.34, 1.83] **** |
| **AB40** | 1.05 [0.92, 1.17] | 0.97 [0.83, 1.11] | 1.20 [1.01, 1.39] | 1.04 [0.90, 1.18] | 1.14 [0.95, 1.33] |
| **AB42** | 1.01 [0.95, 1.07] | 1.01 [0.95, 1.08] | 1.03 [0.93, 1.12] | 0.99 [0.93, 1.06] | 1.05 [0.96, 1.14] |
| **GFAP** | 1.01 [0.93, 1.09] | 1.00 [0.90, 1.10] | 1.03 [0.90, 1.16] | 1.00 [0.90, 1.10] | 1.03 [0.89, 1.16] |
| **NfL** | 1.00 [0.92, 1.09] | 0.92 [0.83, 1.01] | **1.16 [1.04, 1.28] **** | 1.01 [0.92, 1.11] | 1.07 [0.94, 1.19] |
|  |  |  |  |  |  |
| Values shown as mean [95%-CI].  A= derived from a one-sample t-test (reference 1)  B = derived from Generalized Linear Model including sex and weight status as factors and TIB in the night prior to the experiment (as measured via sleep diaries) as a covariate. **P<0.01.  *Abbreviations*: AB, amyloid-beta protein; GFAP, Glial fibrillary acidic protein; NfL, neurofilament light polypeptide; NW, normal-weight; OB, Obesity; pT, Tau-protein | | | | | |

**Supplement 1, Table S2. Raw values for CNS health biomarkers in blood following sleep loss and sleep.**

| **Condition** | **Sleep** | | | | **Sleep loss** | | | |
| --- | --- | --- | --- | --- | --- | --- | --- | --- |
| **Factor** | **Sex** | | **Weight status** | | **Sex** | | **Weight status** | |
|  | **Men** | **Women** | **NW** | **OB** | **Men** | **Women** | **NW** | **OB** |
| **pT231** | 14.0 [10.8 – 17.0] | 9.7 [7.3 – 12.1] | 11.9 [9.9 – 13.8] | 13.1 [7.9 – 18.3] | 17.0 [12.5 – 21.4] | 9.4 [7.2 – 11.5] | 13.0 [10.0 – 16.0] | 16.0 [9.2 – 22.9] |
| **pT181** | 15.7 [11.2 – 20.2] | 12.6 [10.6 – 14.6] | 16.1 [11.7 – 20.5] | 12.0 [9.5 – 14.5] | 21.4 [13.7 – 29.0] | 15.0 [12.0 – 18.1] | 17.0 [11.9 – 22.2] | 22.2 [11.8 – 32.7] |
| **AB40** | 117.5 [99.1 – 135.8] | 88.2 [71.4 – 105.1] | 103.6 [84.8 – 122.5] | 111.3 [91.6 – 131.0] | 108.5 [92.7 – 124.3] | 98.3 [78.0 – 118.6] | 95.1 [81.4 – 108.8] | 120.7 [98.4 – 142.9] |
| **AB42** | 7.8 [6.9 – 8.6] | 6.7 [5.5 – 8.0] | 7.5 [6.5 – 8.4] | 7.2 [6.2 – 8.3] | 7.6 [6.9 – 8.3] | 6.8 [5.6 – 8.0] | 7.2 [6.4 – 8.0] | 7.5 [6.4 – 8.5] |
| **GFAP** | 42.9 [36.4 – 49.5] | 46.6 [37.6 – 55.6] | 45.8 [39.9 – 51.7] | 41.7 [31.5 – 51.9] | 40.7 [35.6 – 45.9] | 46.0 [39.4 – 52.5] | 44.2 [39.3 – 49.1] | 40.2 [33.0 – 47.3] |
| **NfL** | 7.0 [6.0 – 7.9] | 5.9 [4.9 – 7.0] | 7.1 [6.0 – 8.1] | 5.8 [5.2 – 6.4] | 6.3 [5.3 – 7.2] | 6.4 [5.5 – 7.3] | 6.6 [5.6 – 7.6] | 5.8 [5.1 – 6.5] |
|  |  |  |  |  |  |  |  |  |
| Values shown as mean [95%CI].  *Abbreviations*: AB, amyloid-beta protein; GFAP, Glial fibrillary acidic protein; NfL, neurofilament light polypeptide; NW, normal-weight; OB, Obesity; pT, Tau-protein | | | | | | | | |
